# Inferior mesenteric artery embolization ahead of rectal cancer surgery: AMIREMBOL pilot study

**DOI:** 10.1093/bjs/znac071

**Published:** 2022-03-25

**Authors:** Julien Frandon, Laure Berny, Michel Prudhomme, Hélène de Forges, Chris Serrand, Fabien de Oliveira, Jean Paul Beregi, Martin M Bertrand

**Affiliations:** Department of Medical Imaging, Nîmes University Hospital, University of Montpellier, Medical Imaging Group Nîmes (IMAGINE), Nîmes, France; Department of Medical Imaging, Nîmes University Hospital, University of Montpellier, Medical Imaging Group Nîmes (IMAGINE), Nîmes, France; Surgery Department, Nîmes University Hospital, University of Montpellier, Nîmes, France; Department of Medical Imaging, Nîmes University Hospital, University of Montpellier, Medical Imaging Group Nîmes (IMAGINE), Nîmes, France; Department of Biostatistics, Clinical Epidemiology, Public Health, and Innovation in Methodology (BESPIM), CHU Nîmes, Nîmes, France; Department of Medical Imaging, Nîmes University Hospital, University of Montpellier, Medical Imaging Group Nîmes (IMAGINE), Nîmes, France; Department of Medical Imaging, Nîmes University Hospital, University of Montpellier, Medical Imaging Group Nîmes (IMAGINE), Nîmes, France; Surgery Department, Nîmes University Hospital, University of Montpellier, Nîmes, France

## Abstract

Ischaemic conditioning in rectal cancer surgery by preoperative embolization of the inferior mesenteric artery is feasible and safe. It seems to protect from ischaemic stress during the surgical procedure.

## Introduction

Anastomotic leak is a common and feared complication of rectal cancer surgery^1^. It is recognized that good perfusion of the colonic conduit used for the anastomosis is important to reduce the risk of leakage^2,3^. Undertaken a few days before surgery, ischaemic conditioning involves embolizing the vessels planned for ligation during the operation, thereby promoting arterial collateral vessel formation. It has been shown to reduce anastomotic leak rates in oesophageal cancer surgery^4–6^. Advances in endovascular surgery mean that embolization of the inferior mesenteric artery (IMA) to prevent aneurysm endoleaks is feasible and safe^7,8^. The aims of this study were, therefore, to assess the feasibility and safety of IMA ischaemic preconditioning in patients with rectal or sigmoid cancer undergoing surgery, and to evaluate its effect on colonic perfusion.

## Methods

### Patients and study design

Patients aged over 18 years undergoing surgery for rectal or sigmoid cancer were included in this prospective single-centre study. The exclusion criteria were contraindications to surgery or arteriography, superior mesenteric artery (SMA) abnormality or IMA occlusion. The study was approved by an ethical review board (ID RCB 2017-A03527-46, CPP Sud-Méditerrannée II) and registered at ClinicalTrials.gov (NCT0362824). All patients provided signed informed consent. Patients were randomized into two groups to receive either plain arteriography (control group) or endovascular IMA embolization (embolization group). Randomization in a 1 : 1 ratio was performed using SAS^®^ software version 9.2 (SAS Institute, Cary, North Carolina, USA). Patients and surgeons were blinded to the procedure.

### Technical description

#### Arteriography and embolization

Arteriography was performed 3 weeks before surgery by an interventional radiologist with a classical femoral common 4-Fr puncture under local anaesthesia. SMA and IMA diagnostic arteriography was carried out. In the embolization group, the proximal IMA was embolized using coils or plugs to obtain complete IMA occlusion; details are provided in the supplementary material.

#### Surgery

All patients underwent low anterior resection or sigmoid resection with high-tie IMA ligation by a laparoscopic or robot-assisted approach. The resistive index (RI) was used to assess tissue hypoperfusion^9^, and was measured during surgery with an ultrasound probe placed directly on the mesocolon (LA3-16 AD transducer with a Samsung Medison HM70A™ ultrasound system, Seoul, Korea). Systemic arterial BP was recorded before and after IMA ligation.

### Endpoints

The primary endpoint was intraoperative colonic perfusion measured by the RI ((peak systolic velocity – end diastolic velocity)/peak systolic velocity) of the arc of Riolan in the mesocolon, before, immediately after, and 5 min after ligation. After visualization of the arc, several RI measurements were taken until a stable and reproducible tracing was obtained before ligation of the artery. Secondary endpoints were early postembolization complications, assessed during a telephone call on day 5, and surgical complications until hospital discharge graded according to the Clavien–Dindo classification.

### Statistical analysis

Continuous variables are presented as median (range). Values were compared using the Fisher’s exact or Mann–Whitney *U* test. *P* < 0.050 was considered significant.

## Results

### Patients and procedure

From March 2020 to March 2021, 10 patients, of median age 72 (range 53–79) years, were included, with five patients in each group (*Table 1*). Embolization was successful in all five patients, with complete IMA occlusion achieved.

**Table 1:** Patient characteristics and resistive index results

	Embolization group (*n* = 5)	Control group (*n* = 5)	*P*§
Age (years)*	70 (53–71)	74 (63–79)	
**Sex**			
M	2	4	
F	3	1	
BMI (kg/m^2^)*	26 (19–31)	23 (21–28)	
**Cardiovascular risk factors**†	4	4	
**Tumour location**			
Rectum	5	4	
Sigmoid	0	1	
**Preoperative CRT**	4	3	
Procedure duration (min)*	37 (26–72)	13 (4–19)	
Radiation dose (DAP) (mGy/cm^2^)*	281 (120–833)	347 (139–660)	
Duration of hospital stay (nights)*	10 (5–17)	6 (4–25)	
Resistive index*			
Before ligation	0.87 (0.48–0.89)	0.83 (0.71–0.84)	0.53
Immediately after ligation	0.84 (0.37–0.9)	0.5 (0.42–0.75)	0.22
Drop immediately after ligation (%)‡	5.6 (3.4–22.9)	39.0 (9.6–47.0)	0.03
5 min after ligation	0.85 (0.43–0.9)	0.5 (0.45–0.76)	0.22
Drop at 5 min (%)‡	4.5 (1.15–16.7)	36.6 (8.4–42.7)	0.03
Systemic arterial BP (mmHg)*			
Before ligation	76.33 (70.67–87.33)	72.33 (65.00–102.00)	0.83
Immediately after ligation	87.67 (79.33–99.67)	96.67 (69.33–99.67)	0.92
5 min after ligation	75.00 (71.67–90.67)	90.00 (67.00–90.67)	0.83

BMI: Body Mass Index; RI: Resistive Index. *Values are median (range). †Arterial hypertension, diabetes, active tobacco use, hypercholesterolaemia, dyslipidaemia, sedentary lifestyle, personal cardiovascular history, family history of cardiovascular disease. ‡Compared with baseline, before ligation. CRT, chemoradiotherapy; DAP, dose area product. §Mann–Whitney *U* test.

### Colonic perfusion

**Fig. 1:**
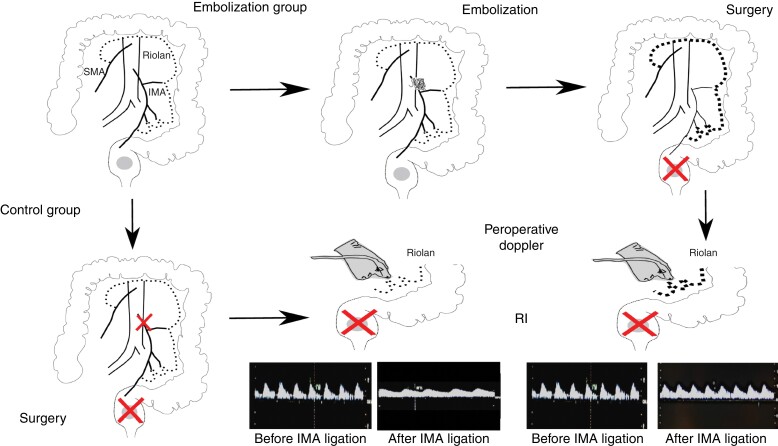
Study protocol In the embolization group, proximal embolization of the inferior mesenteric artery (IMA) (centre top, with the coil in the proximal part of the IMA) was performed 3 weeks before surgery to potentially allow the arc of Riolan (Riolan) to promote arterial collateral vessel formation (bold dotted line). In the control group, plain arteriography was performed but without IMA embolization. Intraoperative Doppler imaging was undertaken directly on the mesocolon and on the arc of Riolan in both groups, and the resistive index (RI) was measured just before surgical ligation (red cross) of the IMA, and at 1 and 5 min after ligation. SMA, superior mesenteric artery.

RI measurement was feasible in all patients. There was a drop in RI immediately after IMA ligation of 39.0 (range 9.6–47.0) per cent in the control group *versus* 5.6 (3.4–22.9) per cent in the embolization group (*P* = 0.032) (*Table 1* and *Fig 1*). This decrease was maintained 5 min after ligation. The arterial BP was similar in the two groups at all times.

### Complications

After arteriography and embolization, no major complications were reported. Minor complications occurred after arteriography in one patient, who developed a localized haematoma at the puncture site. Two patients reported abdominal pain (Clavien–Dindo grade I) 2 days after embolization; both required an analgesia prescription but did not require radiological or surgical intervention and the pain settled spontaneously. After surgery, one patient in the control group sustained an anastomotic leak on day 4 which required a second procedure and longer hospital stay (25 days).

## Discussion

This single-centre randomized study has demonstrated that ischaemic conditioning by preoperative IMA embolization in patients scheduled for surgery to treat rectal or sigmoid cancer is feasible and safe. Improved perfusion in the colonic conduit used for anastomosis was seen in patients who underwent IMA embolization.

The RI is a sonographic index that indicates arterial resistance to flow. It is a common non-invasive tool used to evaluate hepatic^10^ and renal^11^ perfusion. A decrease in RI indicates hypoperfusion in relation to a significant vascular stenosis or occlusion. In this study, the absence of a decrease in RI values in the embolized group reflected the development of vascular collaterality. Other techniques, such as laser Doppler flowmetry^12^ and transanal pulse oximetry^13^, have revealed a similar decrease in colonic blood flow after IMA ligation.

Whether to perform high or low tie of the IMA during left-sided/rectal cancer surgery is debated^14^. In the present study, a high tie was chosen according to the surgical team’s usual clinical practice. Embolization was carried out 3 weeks ahead of surgery; this interval was thought to allow effective preconditioning as previous clinical and experimental studies^15,16^ have shown poorer results with a shorter delay.

The present study has some limitations, including its single-centre pilot design and small size. In addition, follow-up was short and oncological efficacy was not assessed. However, these promising early results should be evaluated in larger multicentre studies that assess the impact of ischaemic preconditioning on the occurrence of postoperative anastomotic leakage.

## Supplementary Material

znac071_Supplementary_Data
