# Supplementary material for: Inferior mesenteric artery embolization ahead of rectal cancer surgery: AMIREMBOL pilot study
Source: Br J Surg. 2022 Mar 25;109(8):650–2. doi: 10.1093/bjs/znac071 (PMC10364674; doi:10.1093/bjs/znac071)
Supplement: znac071_Supplementary_Data [file znac071_supplementary_data.docx]

**Supplementary material**

**Embolization technique**

Both diagnostic and therapeutic arteriographies were performed in a Philips Azurion (Amsterdam, Netherlands) interventional radiology room. The procedure was performed under local anaesthesia (xylocaine 10 mg/mL). The radiological approach was via the right femoral artery using a 4 French femoral introducer, according to the Seldinger technique. Superior mesenteric artery (SMA) catheterism was performed first, in both groups, using a Terumo Glidewire^®^ 0.35 guide and a Cobra II Cordis 4F probe. Iodinated contrast product (Visipaque^TM^ 320 mg I/mL) was then injected automatically (16mL, 4mL/s flow rate). Second, inferior mesenteric artery (IMA) catheterism was performed using a Side 1 Cordis 4F probe. IMA arteriography (9mL, 3mL/s flow rate) allowed the anatomic assessment of the IMA branches and identification of possible anatomical variants.

In the “embolization” group, proximal embolization of the IMA was also performed, upstream of the IMA dividing branches, either by plug or coil, at minimum 1 cm after the IMA ostia as to allow IMA ligation during surgery. The proximal obstruction efficacy was checked by second injection at the IMA ostia (9mL, 3mL/s flow rate), ensuring no contrast product flowed across the coils or plugs.

Plugs (patient 2 and patient 5: biconic nitinol vascular plugs, Amplatzer^TM^ type IV, 4 and 5 mm diameter) or coils (patient 1 with controlled-release fibrous macrocoils, Retracta®, 6 x 70 mm (x2); patients 3 and 4 using two pushable fibrous microcoils, Nester®, 3 mm x 14 cm) were used for embolization at the discretion of the operator.(Supplementary Figure 1).

No premedication and no preventive analgesic treatment was given.


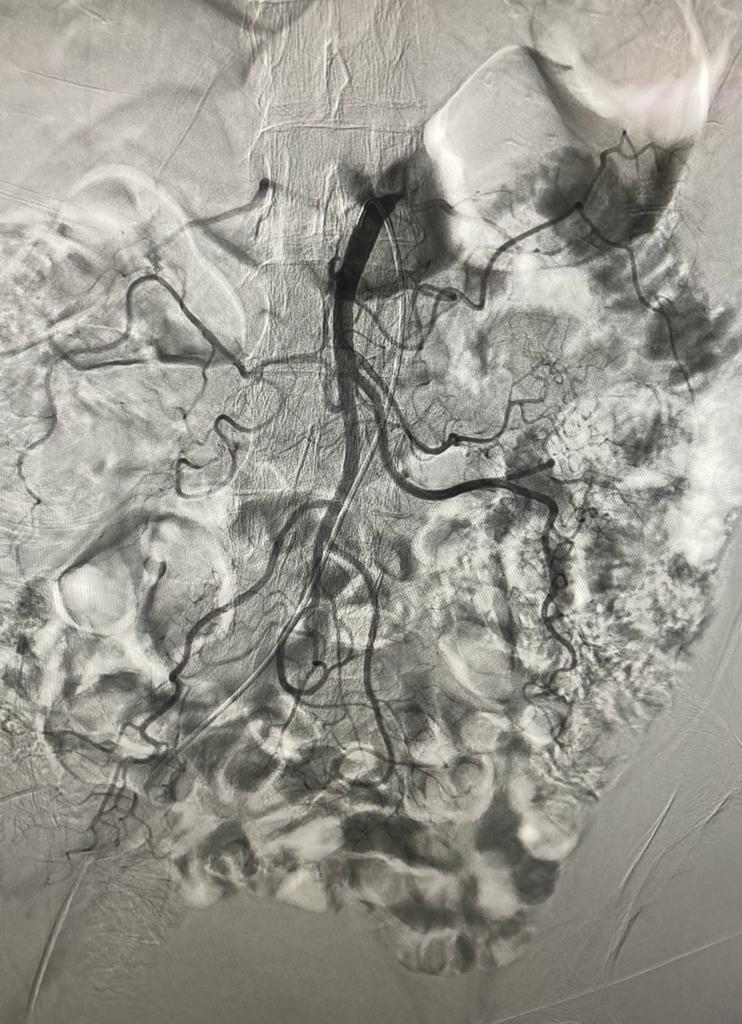

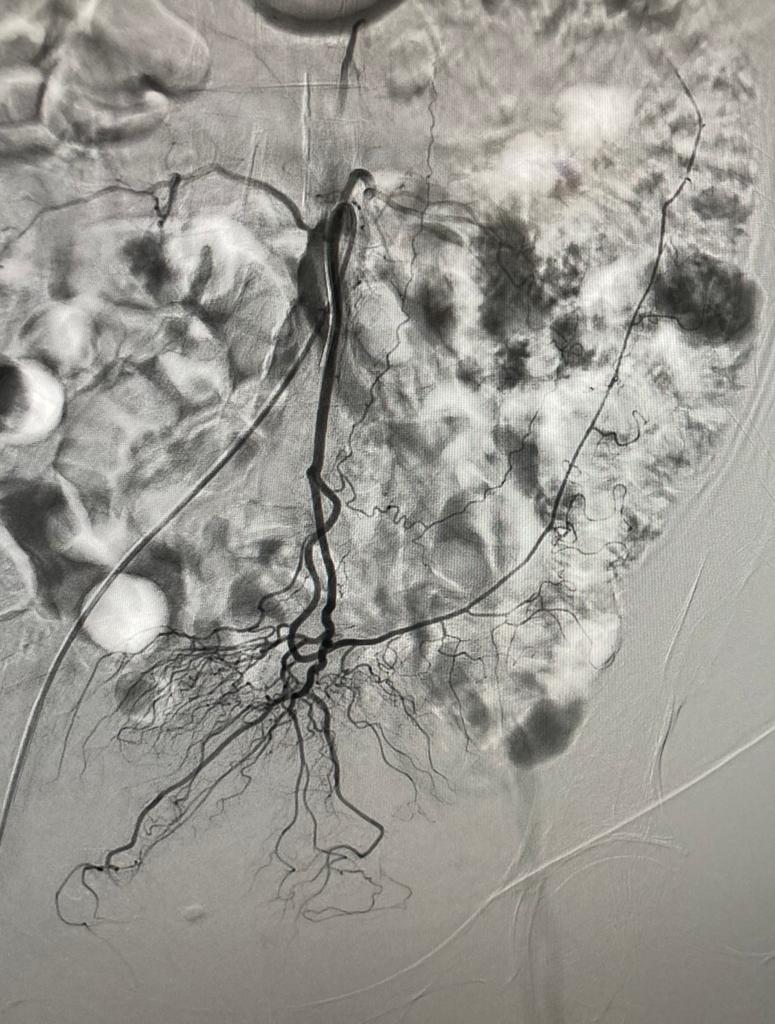

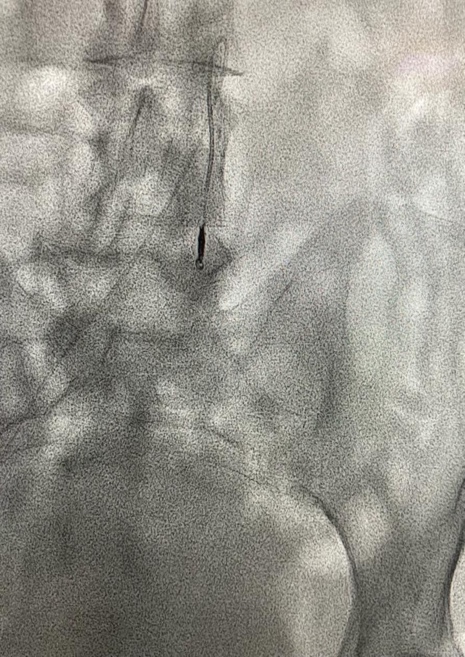

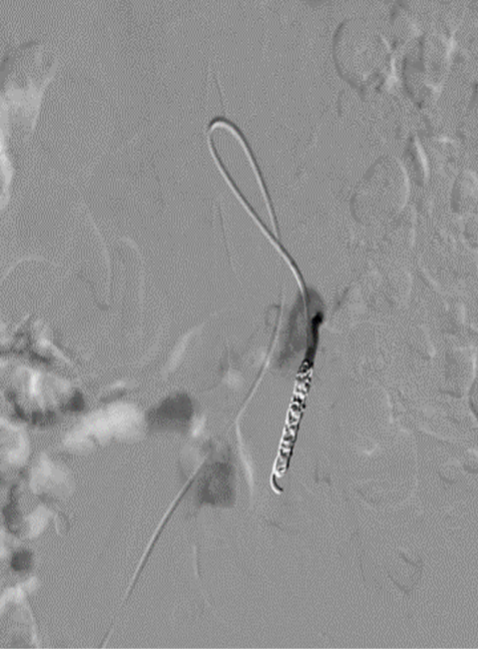

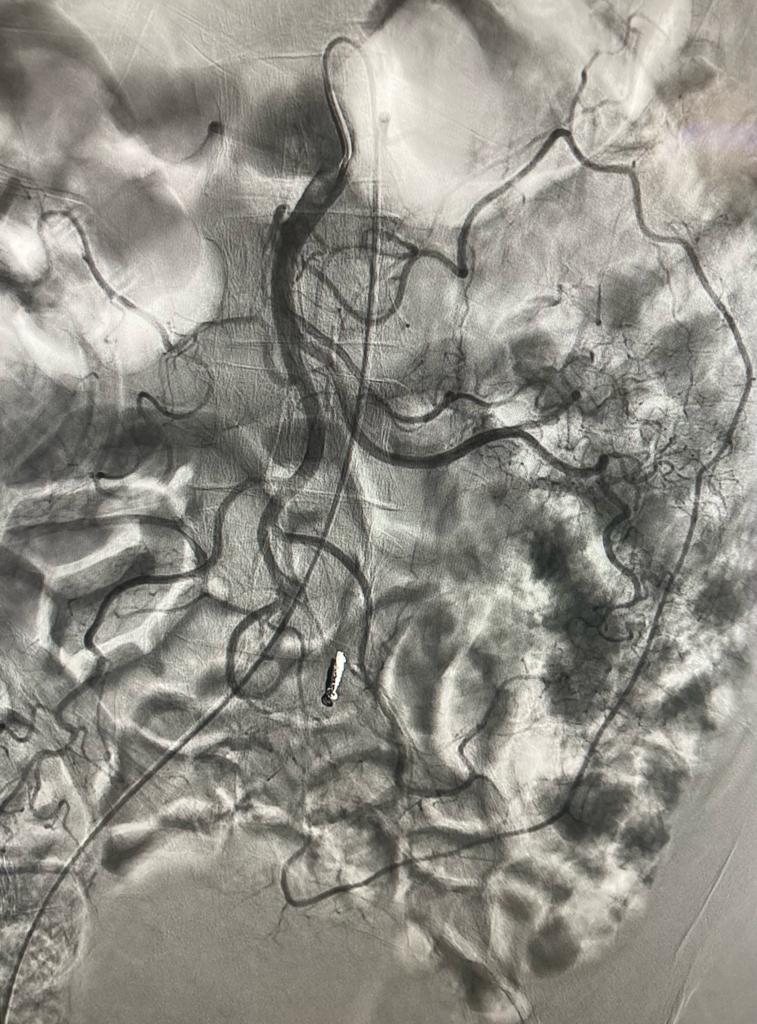

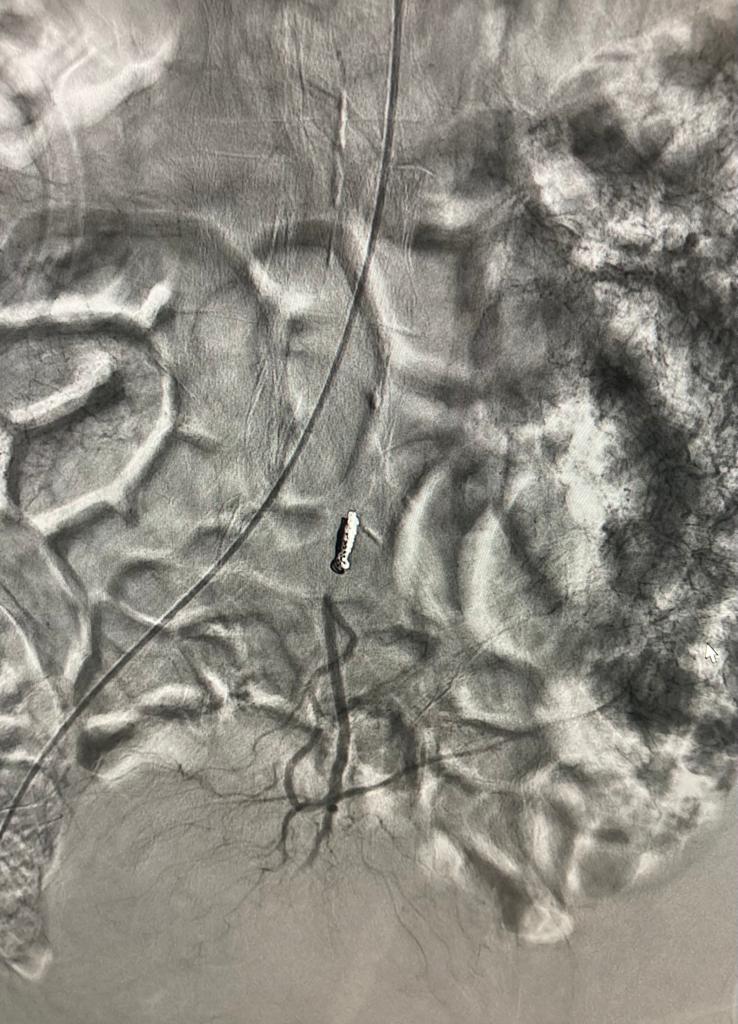


f

d

c

b

a

e

**Figure S1.** Example of initial digital substracted arteriographies of the SMA (a) and the IMA (b). Middle colic artery (a) and left colic artery (b) are pointed out with white arrows. Fluoroscopy showing IMA embolization with coils (white asterix) (c). Embolization was checked to be efficient, with no flow of contrast product downstream the embolization device on digital substracted angiography from AMI (d). Final SMA arteriography after AMI embolization of AMI with early (e) and delayed (f) acquisitions. The expansion of the Arc of Riolan is shown by white arrow heads (e and f). Embolization efficiency was also confirmed by the development of collateral vascularity and the expansion of the Arc of Riolan with revascularization downstream the embolization device (f).
